# Storax, A Promising Botanical Medicine for Treating Cardio-Cerebrovascular Diseases: A Review

**DOI:** 10.3389/fphar.2021.785598

**Published:** 2021-11-30

**Authors:** Zhuo Xu, Danni Lu, Jianmei Yuan, Mihong Ren, Rong Ma, Qian Xie, Yong Li, Jinxiu Li, Jian Wang

**Affiliations:** State Key Laboratory of Southwestern Chinese Medicine Resources, School of Pharmacy, Chengdu University of Traditional Chinese Medicine, Chengdu, China

**Keywords:** storax, cardio-cerebrovascular diseases, chemical composition, pharmacological activities, cerebral and cardiac treatment synchronously

## Abstract

In recent years, the incidence and mortality of cardio-cerebrovascular diseases have been increasing year by year, which has become global burden and challenge. Based on the holistic thinking of “brain disease affects the heart” and “heart disease affects the brain,” as well as the characteristics of multi-target and multi-path effects of Chinese medicine, Chinese medicine is more advantageous in the treatment of cardio-cerebrovascular diseases. As a botanical medicine, storax is known for its resuscitation, filth avoidance and pain-relieving effects in the treatment of cardio-cerebrovascular diseases. By reviewing and collating the relevant domestic and international literature in the past 10 years, we have sorted out an overview of the medicinal parts, traditional uses and chemical composition of storax. For the first time, based on the idea of “cerebral and cardiac simultaneous treatment,” the pharmacological activities and mechanisms of heart and brain protection of storax for treating cardio-cerebrovascular diseases were summarized and analyzed, showing that storax has the pharmacological effects of anti-cerebral ischemia, regulation of blood-brain barrier, bidirectional regulation of the central nervous system, anti-myocardial ischemia, anti-arrhythmia, anti-thrombosis and anti-platelet aggregation. It mainly exerts its protective effects on the brain and heart through mechanisms such as inhibition of inflammatory immune factors, anti-oxidative stress, anti-apoptosis, pro-neovascularization and regulation of NO release. On the basis of the current findings and limitations, the future research strategies and perspectives of storax are proposed, with a view to providing a reference for further application and development of this medicine, as well as contributing new thoughts and visions for the clinical application of “treating brain-heart synchronously”.

## 1 Introduction

Storax (ST), which is also named Styrax or Su-Hexiang, is a kind of processed and refined aromatic resin exuded from the trunk of the *Liquidambar orientalis* Mill in the family Hamamelidaceae (State Pharmacopoeia Committee, 2020). It has been not only widely used as a folk medicine in East Asia, India, Africa and Turkey for many years (U.S. Pharmacopeial Convention, 2017; Duru et al., 2010), but also being a popular spice for food and wine (Zhang et al., 2020). During the Qin and Han dynasties, ST was introduced to China by the Silk Road and was widely used in healthcare and social life. Due to its high safety profile and beneficial effects on various diseases, ST is used as a crucial ingredient in the preparation of various traditional Chinese prescriptions, for example, Guanxin Suhe Pill, Shexiang Baoxin Pill, Su Bing Dripping Pill and ST Pill. Nowadays, Chinese medicine compounds containing ST are widely used in Asian countries for the prevention and treatment of cardio-cerebrovascular diseases (CCVDs) (Xu, 2014; Li et al., 2016; Zhou et al., 2016; Lu et al., 2018; Zou et al., 2020).

CCVDs are ischaemic or hemorrhagic diseases of the heart and brain caused by thickened blood, atherosclerosis, hypertension and hyperlipidemia, including cardiovascular and cerebrovascular lesions, which pose a serious threat to human health. According to Global Burden of disease 2016, there were approximately 80.1 million people with cerebrovascular disease worldwide, of which 67.6 million had ischemic stroke and 2.7 million died (Benjamin et al., 2019). Similar to ischemic stroke, the incidence of coronary heart disease was high, too. In 2016, it was estimated that 17.9 million people died from cardiovascular disease, accounting for 31% of deaths around the world (Benjamin et al., 2019). Therefore, in order to prevent and treat these diseases, it is urgent to identify the pathogenesis of CCVDs and clarify the relationship between cardiovascular diseases and cerebrovascular diseases, so as to find medications with better efficacy and fewer side effects.

**FIGURE 1:**
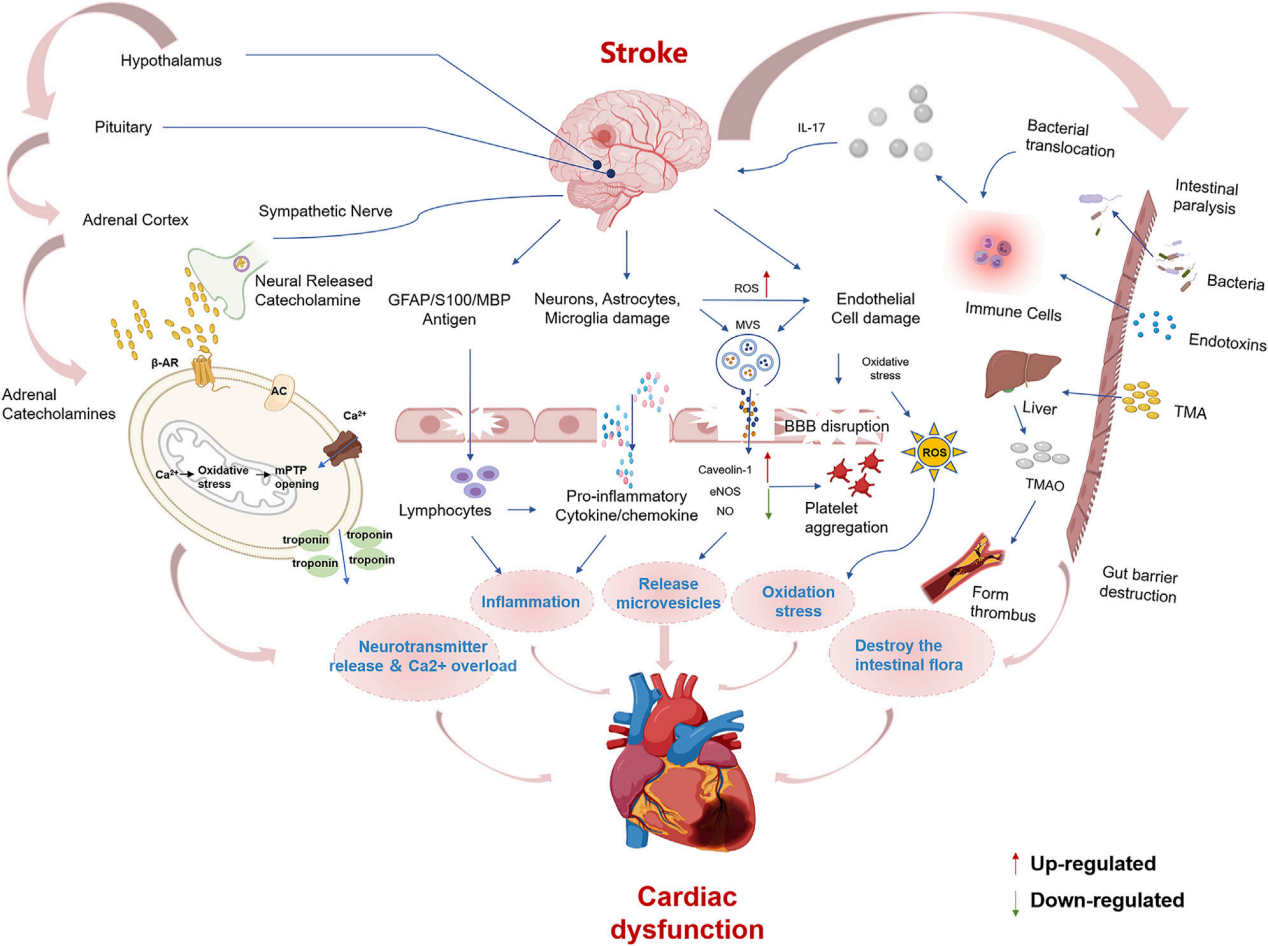
Summary of mechanisms for brain-heart association after stroke Cardiac dysfunction after stroke may be caused by the following mechanisms, including ① Activation of the hypothalamic-pituitary-adrenal axis and sympathetic modulation cause a surge of catecholamines, which act on β-receptors. β-receptor coupling stimulates G proteins to activate adenylate cyclase (AC), causing mitochondrial Ca^2+^ overload and failure of ATP synthesis leading to cardiomyocyte death. ② Brain cells that die after stroke release damaging antigens that can enter the body circulation through the ruptured blood-brain barrier, and the antigen-specific autoimmune response leads to the secretion of pro-inflammatory cytokines. Damaged neurons, microglia, endothelial and astrocytes, and the spleen can stimulate the release of pro-inflammatory cytokines and chemokines. ③ Microvesicles (MVs) released from damaged astrocytes, neurons and microglia after stroke inhibit eNOS function and increase Caveolin-1 while decreasing NO synthesis, leading to endothelial dysfunction and platelet aggregation. ④ Elevated reactive oxygen species and endothelial cell damage lead to oxidative stress. ⑤ Stroke increases the permeability of the intestinal barrier, causing dysbiosis of the intestinal flora and translocation of bacteria and endotoxin into the bloodstream. TMAO is a hepatic oxidation product of the microbial metabolite TMA. TMAO induces thrombosis and atherosclerosis formation.

According to traditional Chinese medicine, human beings are an organic whole. Although the “brain” and “heart” are two different organs, they are pathologically related to each other, and CCVDs often exist at the same time. The incidence of stroke has been reported to be more than double in patients with coronary heart disease, more than triple in those with hypertension, quadruple in those with heart failure, and quintuple in those with atrial fibrillation (Chen et al., 2017). Likewise, stroke (cerebral ischemia, cerebral hemorrhage, or subarachnoid hemorrhage) can lead to cardiac insufficiency, arrhythmias, and even heart failure ultimately. The interaction of the brain and heart after stroke is described in Figure 1. Cardiac dysfunction can be induced after stroke by modulating the hypothalamic-pituitary-adrenal axis, activating sympathetic and parasympathetic nerves to release catecholamine (Vaseghi et al., 2017; Veltkamp et al., 2019), disrupting the gut microbiota (Nam 2019), inducing immune response (Zhao et al., 2019) and inflammation (Kelly et al., 2021) as well as releasing microvesicles (Huo et al., 2021). Hence, it is reasonable and inevitable to adopt the methodology of “regulating the brain and heart simultaneously,” and the flexible application of this principle can help improve the curative effect of CCVDs. At present, 84 chemical components have been isolated from ST, mainly including terpenoids, aromatic organic acids, and their derivatives, which have various pharmacological activities (e.g., anticoagulant, anti-inflammatory and antibacterial).

Until recently, researchers at home and abroad have achieved great findings on CCVDs. However, there has been no published systematic and comprehensive review that focuses on various important aspects of ST in the treatment of CCVDs. In this paper, with “Storax,” “Styrax” and “Su-Hexiang” as keywords, the medicinal parts, traditional usages, phytochemistry, and pharmacological activities of ST in the past 10 years are summarized by searching the relevant documents in Wan Fang, China National Knowledge Infrastructure, Web of Science, PubMed and other databases. The limitations of current works are also debated, coupled with prospects on the future research directions of ST, which are paramount for deeper exploration, development and utilization of ST, and will offer a new outlook for the clinical diagnosis and treatment of CCVDs, besides supplying the basis for the application of the theory of “simultaneous regulation of the brain and heart.”

## 2 Medicinal Parts of ST

**FIGURE 2:**
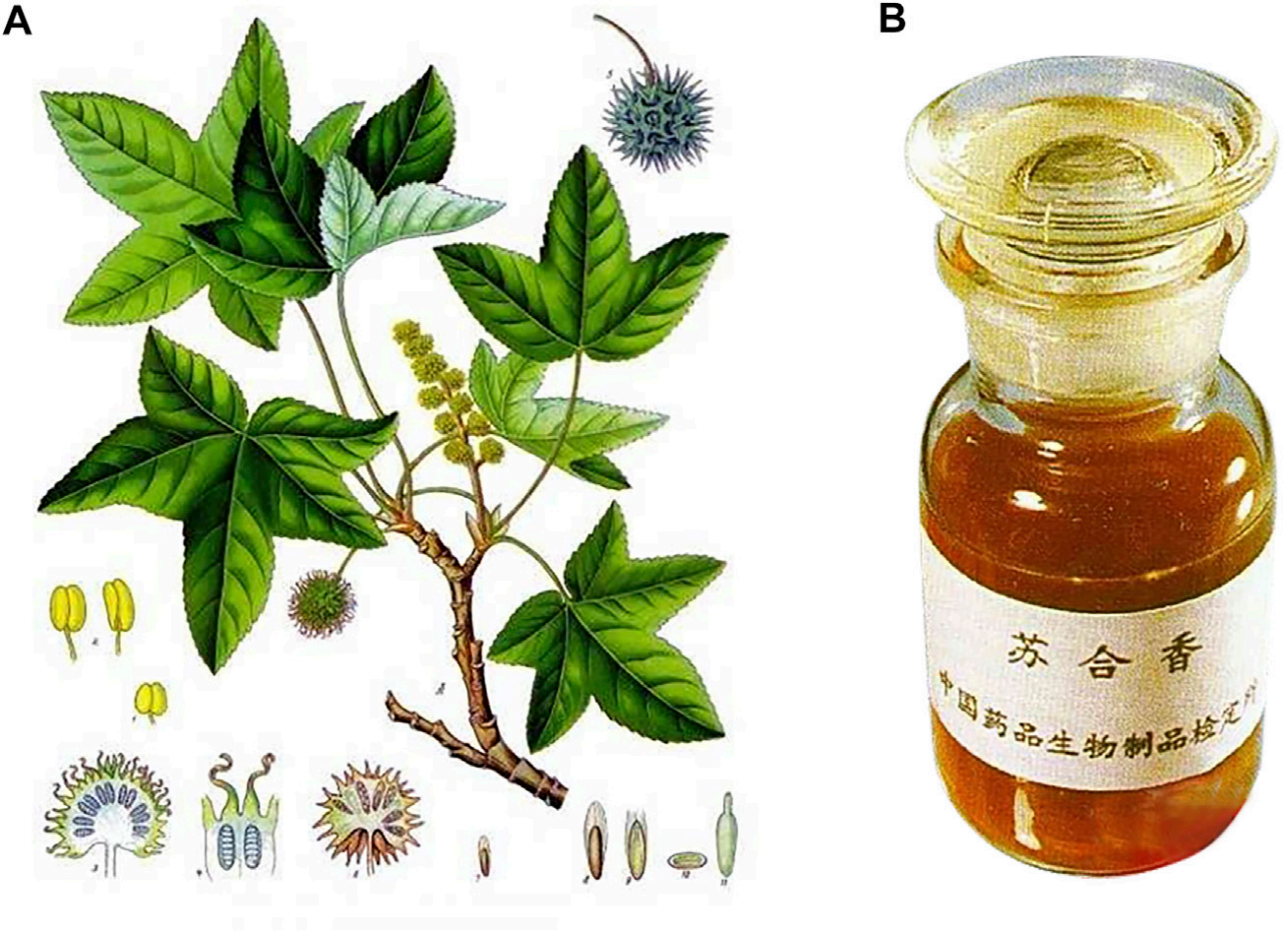
Branches of storax **(A)**; storax oil **(B)** (https://image.baidu.com/).

*Liquidambar orientalis* Mill. is a tree whose branches and leaves are shown in Figure 2A. The plant is native to southern Asia Minor, in particular, Turkey and northern Syria. Nowadays there are several introductions in Guangxi Province, China. The bark is usually struck or broken in early summer to reach the wood so that the resin is secreted and seeped into the bark. In autumn, the injured bark is peeled off and used to extract the resin. The remaining residue is boiled in water and pressed again, then filtering out the impurities and extracting the balsam as ordinary ST. The balsam is dissolved in ethanol, filtered and steamed out of the ethanol. Then it becomes refined balsam, also known as ST oil or flowing ST, characterized as semi-fluid, brown or dark brown, translucent, viscous, aromatic, shown in Figure 2B (Editorial Board of the State Administration of TCM, 1998).

## 3 Traditional Usages

ST is widely used in both Asia and Africa, not only as the main element in the formulation of numerous Chinese medicine prescriptions but also as a prevalent spice in the manufacture of food and wine (Zhang et al., 2020). In Turkey, in addition to using ST to preparing medicines, it is also applied in the production of perfume, which is one of Turkey’s export commodities (Kültür, 2007). In Turkish folk medicine, ST is also utilized to treat varied ailments, for instance, cleft lips, burns, peptic ulcers, nocturnal enuresis, parasitic infections, and as an antiseptic or phlegmatic agent (Gurbuz et al., 2013). For the treatment of peptic ulcers, the mixture of ST and honey (1:5 ratio) is favored by the population (Honda et al., 1996). It was also used in ancient Egypt for mummification.

The usage of ST in medicine has been recorded since the Southern and Northern Dynasties in China. “Long-term intake is good for the spirit and makes the body lighter and healthier so that people live longer” was recorded in “*Mingyi Bielu*” compiled by Hongjing Tao (456–536 CE) during the Wei and Jin Dynasties, describing the medicinal value of ST for the first time. “*Compendium of Materia Medica*” authored by Shizhen Li (1518-1593 CE), stated that “ST, the aroma of which is able to move and flow though all the orifices and organs, so it can ward off all pathogenic qi.” According to another Chinese ancient classical book “*Bencao Biandu*” in the Qing Dynasty, the fragrance of ST is stronger than all kinds of incense, warm in nature and non-toxic, as soon as attributing to heart and spleen meridian, can be inducing resuscitation and expelling the pathogens as well. As a result, all pathological conditions with the inner invasion of pathogens, let’s say stroke, phlegm stroke, and qi stroke can use it to treat. Currently, ST is considered to have aromatic resuscitation, warming the heart, dispersing cold and relieving pain effects. It is frequently used for stroke, heatstroke, phlegm coma, heart and stomach pains, suggesting a therapeutic effect on chest paralysis and cerebral stroke. Presently, the fat-soluble components of ST oil are also extracted to obtain a water-insoluble perfume, which is incorporated into lavender recipes to provide a lingering effect, similar to “fragrance fixative.”

In traditional Chinese medicine applications, ST is often combined with musk and borneol, such as ST Pill from “*Taiping Huimin Heji Ju Fang*” in Song Dynasty. It is a common formula for the clinical treatment of angina pectoris in coronary heart disease and cerebral ischemia. Modern scientists have simplified and created new preparations based on this formula, such as Guanxin Suhe Pill (Capsule) and Su Bing Dripping Pill, which are commonly used for coronary heart disease and angina pectoris. Fangfei Zhang used ST Pill to treat abdominal pain, hypochondriac pain, and pinnacle headaches that were caused by qi stagnation, blood stasis, and cold clotting, with remarkable grades (Zhao et al., 2006). ST mixed with olive oil and applied externally to treat scabies (Editorial Board of the State Administration of TCM, 1998). Topical application of ST is effective in the reduction of eczema and itchiness by relieving inflammation and promoting the healing of ulcers, suggesting its efficacy in the treatment of skin conditions (Editorial Board of the State Administration of TCM, 1998). Simplified and improved from ST Pill, Shexiang Baoxin Pill and Guanxin Suhe Pill are commonly used in clinical practice for the treatment of CCVDs. Guanxin Suhe Capsule has the effect of regulating qi, broadening the chest and reducing soreness, mainly treating chest congestion and breath-holding. It is commonly used clinically for the prevention and treatment of angina pectoris with precise efficacy, increasing coronary flow and improving microcirculation, also lowering blood lipids and raising high-density lipoprotein, and has no adverse effects on liver and kidney. Wei Wan et al. compared the efficacy of Guanxin Suhe Pill with that of Suxiao Jiuxin Pill and found that Guanxin Suhe Pill was slightly more effective than Suxiao Jiuxin Pill, and there was no significant difference in the onset of action between the two, but the duration of Guanxin Suhe Pill was significantly longer than that of Suxiao Jiuxin Pill (Wan and Hang, 2005). Ma et al. used ST Pill in combination with conventional western medical measures to treat acute heavy craniocerebral injury identified as qi blockage of the brain, and the efficacy was significantly better than conventional western medical treatment, effectively reducing the disability rate and the incidence of complications (Ma and Xie, 2008).

## 4 Phytochemical Constituents

Despite its long history of use as a botanical, the systematic study of the chemical composition of ST has started relatively late. ST consists mainly of resin (about 36%), water (about 14–21%), and oily liquid. To date, 84 compounds have been isolated and identified from ST, and the composition obtained by different extraction methods is not entirely consistent. Among them, aromatic compounds and terpenoids are the most important active components of ST, which are considered to be important for future evaluation of medicinal efficacy.

### 4.1 Aromatic Compounds

**FIGURE 3:**
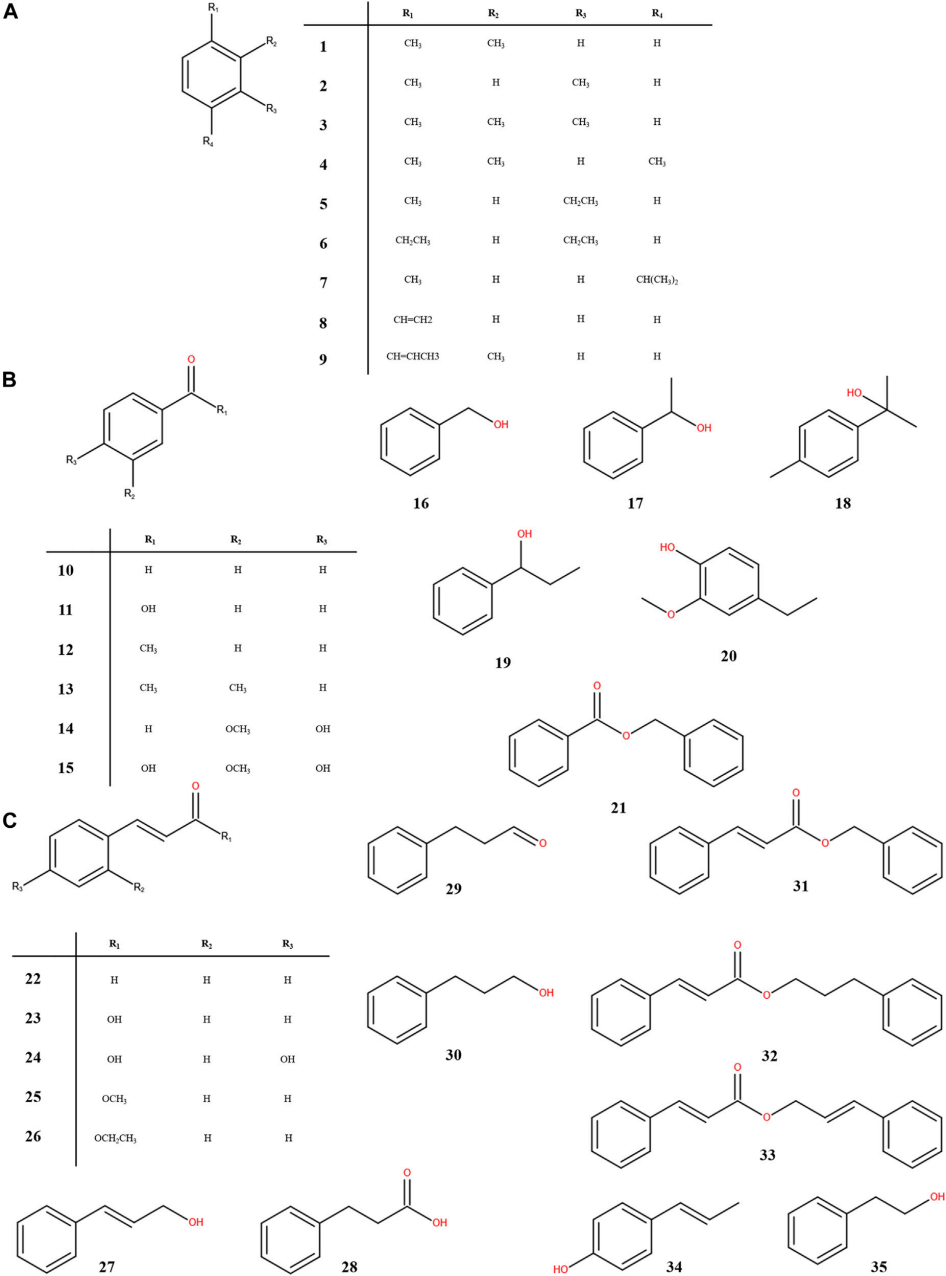
Chemical structures of aromatic compounds from storax. Aromatic hydrocarbons **(A)**; aromatic acids and their derivatives with the skeleton of benzoic acid **(B)** or cinnamic acid **(C)** as the core structure.

At present, 35 aromatic compounds (1–35) have been isolated from ST as shown in Table 1, with physiological activities such as anti-myocardial ischemia, anti-cerebral ischemia and anti-thrombotic. These include nine aromatic hydrocarbon compounds (1–9) as shown in Figure 3A, and a total of 26 aromatic organic acids and their derivatives (10–35) have been identified, which have benzoic acid (Figure 3B) and cinnamic acid (Figure 3C) skeletons as their parent structures, respectively. Among them, cinnamic acid is an index component for the determination of the content of ST in the 2020 edition of the Chinese Pharmacopoeia and may also be one of the main components for its pharmacological action (State Pharmacopoeia Committee, 2020). Cinnamic acid has been shown to have a cardioprotective effect on isoproterenol-induced acute myocardial ischemia models (Song et al., 2013). Furthermore, cinnamic acid activates PPARα to stimulate lysosomal biogenesis to reduce amyloid plaque pathology in mice models of alzheimer’s disease, thus demonstrating its cerebral protective effect (Chandra et al., 2019).

**TABLE 1:** Chemical constituents of aromatic compounds from Storax.

Classification	NO.	Name	References
Aromatic hydrocarbons	1	o-xylene	Peng et al. (2012)
	2	m-Xylene	Peng et al. (2012)
	3	1,2,3-Trimethylbenzene	Peng et al. (2012)
	4	1,2,4-Trimethylbenzene	Peng et al. (2012)
	5	3-Ethyltoluene	Peng et al. (2012)
	6	m-Diethylbenzene	Peng et al. (2013)
	7	4-Isopropyltoluene	(Wang et al., 2012b; Gurbuz et al., 2013; Peng et al., 2013)
	8	Styrene	(Gurbuz et al., 2013; Park 2014)
	9	1-Allyl-2-methyllbenzene	(Wang et al., 2012b; Peng et al., 2013)
Aromatic organic acids and their derivatives	10	Benzaldehyde	(Wang et al., 2012b; Gurbuz et al., 2013; Peng et al., 2013; Park 2014)
	11	Benzoic acid	(Wang et al., 2012b; Huang et al., 2019)
	12	Acetophenone	(Wang et al., 2012b; Gurbuz et al., 2013; Peng et al., 2013; Park 2014)
	13	3-Methylacetophenone	Peng et al. (2013)
	14	Vanillin	Wang et al. (2011)
	15	Vanillic acid	Wang et al. (2011)
	16	Benzyl alcohol	(Wang et al., 2012b; Gurbuz et al., 2013; Park 2014)
	17	1-phenyl-1-ethanol	Park (2014)
	18	2-(4-Methylphenyl)propan-2-ol	(Wang et al., 2012b; Peng et al., 2013)
	19	1-phenyl-1-propanol	Gurbuz et al. (2013)
	20	4-Ethylguaiacol	Gurbuz et al. (2013)
	21	Benzyl benzoate	(Peng et al., 2013; Huang et al., 2019)
	22	Cinnamaldehyde	(Wang et al., 2012b; Gurbuz et al., 2013; Peng et al., 2013)
	23	Cinnamic acid	Huang et al. (2019)
	24	p-Hydroxycinnamic acid	Wang et al. (2011)
	25	(Z)-Methyl cinnamate, (E)-Cinnamaldehyde	Gurbuz et al. (2013)
	26	(E) -Ethyl cinnamate	Gurbuz et al. (2013)
	27	Cinnamyl alcohol	(Wang et al., 2012b; Gurbuz et al., 2013; Park 2014)
	28	Hydrocinnamic acid/3-Phenylpropanoic acid	(Wang et al., 2012b; Park 2014)
	29	Phenylpropyl aldehyde; 3-Phenylpropanal	Gurbuz et al. (2013)
	30	3-Phenyl-L-propanol	(Wang et al., 2012b; Peng et al., 2012)
	31	Benzyl cinnamate	Huang et al. (2019)
	32	3-phenylpropyl cinnamate	(Wang et al., 2020b; Zhang et al., 2020)
	33	Cinnamyl cinnamate	(Huang et al., 2019; Wang et al., 2020b; Zhang et al., 2020)
	34	Phenol	Gurbuz et al. (2013)
	35	Phenyl ethyl alcohol	Gurbuz et al. (2013)

### 4.2 Terpenoids

**FIGURE 4:**
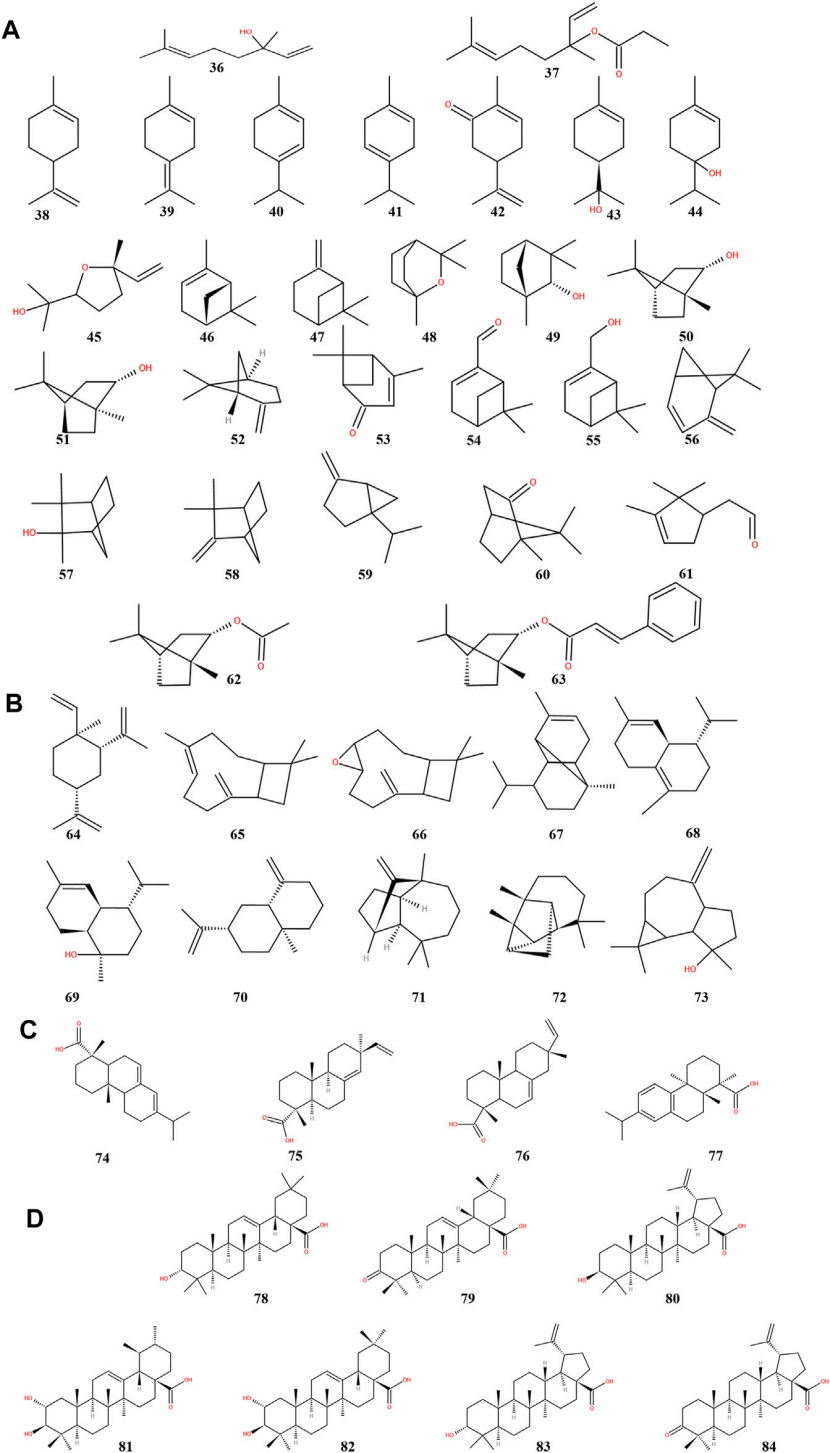
Chemical structures of terpenoids from storax. Monoterpenoids **(A)**, sesquiterpenoids **(B)**, diterpenoids **(C)** and pentacyclic triterpenoids **(D)**.

ST is rich in terpenoids (Table 2), which have pharmacological effects such as anti-inflammatory, hypolipidemic and hypotensive, and are classified by structure into monoterpenes (36–63) (Figure 4A), sesquiterpenes (64–73) (Figure 4B), diterpenes (74–77) (Figure 4C) and pentacyclic triterpenes (78–84) (Figure 4D). These seven pentacyclic triterpenoids including oleanolic acid (78), oleanolone acid (79), betulinic acid (80), corosolic acid (81), maslinic acid (82), epibetulinic acid (83) and betulonic acid (84) are not only widely used as food additives, but also efficiently inhibit Human carboxylesterase 1A1 (hCES1A) and Pancreatic lipase (PL) activities (Wang et al., 2020b; Wang et al., 2020c; Zhang et al., 2020). Thereby ST could be a potential drug for the treatment of obesity and obesity-related metabolic disorders such as stoke and coronary heart disease, which is a hot research topic at the moment. Furthermore, oleanolic acid has been shown to have neuroprotective effects in rats with subarachnoid hemorrhage via SIRT1-mediated deacetylation of HMGB1 (Han et al., 2021). At the same time, oleanolic acid pretreatment also reduce ischemia-reperfusion injury in mice, reflecting its cerebroprotective effect (Shi et al., 2021). Betulinic acid has also been shown to reduce brain injury not only by reducing oxidative stress but also by activating the SIRT1/FoxO1 pathway to inhibit autophagy and ameliorate brain injury in middle cerebral artery occlusion (MCAO) rats (Zhao et al., 2021). Corosolic acid plays a cardioprotective role against dox-induced cardiotoxicity by restoring autophagic flux and improving mitochondrial function (Che et al., 2021). Maslinic acid ameliorates isoprenaline-induced myocardial necrosis by inhibiting the free radical generating enzyme XO (Shaik et al., 2021). All these findings suggest that terpenoids may exert protective effects in the heart and brain and may be the main medicinal substances of ST.

**TABLE 2:** Chemical constituents of terpenoids from storax.

Classification	NO.	Name	References
Monoterpenes	36	Linalool	Gurbuz et al. (2013)
	37	Linalyl Propionate	Peng et al. (2013)
	38	limonene	(Wang et al., 2012b; Gurbuz et al., 2013)
	39	Terpinolene	Peng et al. (2013)
	40	α-Terpinene	Peng et al. (2013)
	41	γ-Terpinene	(Wang et al., 2012b; Gurbuz et al., 2013; Peng et al., 2013)
	42	Carvone	(Wang et al., 2012b; Peng et al., 2012; Peng et al., 2013)
	43	(-)-α-Terpineol	Gurbuz et al. (2013)
	44	Terpinen-4-ol	(Peng et al., 2012; Gurbuz et al., 2013)
	45	Linalool oxide (Furanoid)	(Wang et al., 2012b; Gurbuz et al., 2013; Peng et al., 2013)
	46	α-Pinene	(Wang et al., 2012b; Peng et al., 2012; Gurbuz et al., 2013; Peng et al., 2013; Park 2014)
	47	β-pinene	(Wang et al., 2012b; Peng et al., 2012; Gurbuz et al., 2013; Peng et al., 2013; Park 2014)
	48	1,8-cineole	(Gurbuz et al., 2013; Peng et al., 2013)
	49	(+)-Fenchol	Gurbuz et al. (2013)
	50	Borneol	(Wang et al., 2012b; Peng et al., 2012; Gurbuz et al., 2013)
	51	Exoborneol	Peng et al. (2013)
	52	(-)-trans-pinocarveol	Peng et al. (2012)
	53	(S)-verbenone/4, 6, 6-Trimethyl-bicyclo [3.1.1] hept-3-en-2-one	(Wang et al., 2012b; Peng et al., 2012)
	54	Myrtenal	(Peng et al., 2012)
	55	Myrtenol	(Wang et al., 2012b; Peng et al., 2012)
	56	Verbenene	Peng et al. (2013)
	57	Camphene hydrate	Gurbuz et al. (2013)
	58	Camphene	Wang et al. (2012b)
	59	Sabinene	Peng et al. (2013)
	60	(±)-Camphor	(Peng et al., 2012; Peng et al., 2013)
	61	Campholenaldehyde	Peng et al. (2013)
	62	Bornyl acetate	(Wang et al., 2012b; Gurbuz et al., 2013; Peng et al., 2013)
	63	Bornyl cinnamate	(Wang et al., 2012b; Peng et al., 2012)
Sesquiterpenoids	64	beta-elemene	Gurbuz et al. (2013)
	65	(-)-β-caryophyllene	(Wang et al., 2012b; Peng et al., 2012; Peng et al., 2013; Park 2014)
	66	Caryophyllene Oxide	Peng et al. (2012)
	67	α-copaene	(Wang et al., 2012b; Gurbuz et al., 2013; Peng et al., 2013)
	68	δ-cadinene	Gurbuz et al. (2013)
	69	δ-Cadinol	Gurbuz et al. (2013)
	70	β-Selinene	Peng et al. (2012)
	71	(+)-Longifolene	(Wang et al., 2012b; Peng et al., 2012; Peng et al., 2013)
	72	(+)-longicyclene	Peng et al. (2013)
	73	(+)Spathulenol	Peng et al. (2013)
Diterpenoids	74	Abietic acid	Huang et al. (2019)
	75	Pimaric acid	Wang et al. (2011)
	76	(5ξ,9ξ,13α)-Pimara-7,15-dien-18-oic acid	Wang et al. (2011)
	77	dehydroabietic acid	(Wang et al., 2011; Huang et al., 2019)
Pentacyclic triterpenoids	78	Oleanic acid	(Wang et al., 2011; Wang et al., 2020b; Wang et al., 2020c; Zhang et al., 2020)
	79	Oleanonic acid	(Wang et al., 2011; Wang et al., 2020b; Wang et al., 2020c; Zhang et al., 2020)
	80	Betulinic acid	(Wang et al., 2020b; Wang et al., 2020c; Zhang et al., 2020)
	81	Corosolic acid	(Wang et al., 2020b; Wang et al., 2020c; Zhang et al., 2020)
	82	Maslinic acid	(Wang et al., 2020b; Wang et al., 2020c; Zhang et al., 2020)
	83	Epibetulinic acid	(Wang et al., 2020b; Wang et al., 2020c; Zhang et al., 2020)
	84	Betulonic acid	(Wang et al., 2020b; Wang et al., 2020c; Zhang et al., 2020)

## 5 Pharmacological Activities

As ST is mainly used clinically for the treatment of CCVDs, there is a wealth of research data on its pharmacological action in recent years, which is also the main focus of this review. In accordance with the general requirements of phytopharmacology research (Heinrich et al., 2020), we observed type of extract used, the model used, the dose range tested, duration, the minimal active dose, if and what controls were used as shown specifically in Tables 3, 4. In addition, it also has assorted pharmacological effects, e.g., antibacterial (Wang et al., 2010; Wang et al., 2011), insecticidal (Imam, 2013), antidepressant (Min et al., 2018), inhibiting abnormal excitation of uterine smooth muscle (Wang et al., 2014), anti-prostate cancer cells (Atmaca et al., 2020), promoting wound healing (Ocsel et al., 2012) and hepatoprotective (Suzek et al., 2016), which will not be discussed in this article.

**TABLE 3:** Pharmacological effects of storax on cerebrovascular diseases (“↓,” decrease; “↑,” increase).

Pharmacological activities	Extract	Model/Method	Dose/Duration	Minimal active dose	Control		Results	References
					Positive	Negative		
Anti-cerebral ischemia	Volatile oil	PC12 cells/*In vitro*	1.25, 2.5, 3.75 μg/ml/24 h	1.25 μg/ml	NA	High glucose DMEM culture with 10% FBS	Cell viability, NO ↑; Excessive inward flow of Ca^2+^, Ca^2+^ overload-related injury ↓	(Liu et al., 2010b)
	Volatile oil	PC12 cells/*In vitro*	20, 40, 80 μg/ml/1 day	20 μg/ml	NA	High glucose DMEM culture with DMSO	Ischemic-hypoxic injury, Ca^2+^ inward flow ↓	Wang et al. (2012a)
	Whole medicine	KM mice/*In vivo*	1.332 g/kg/3 days	1.332 g/kg	NA	5% Tween +0.2% CMC-Na	Number of mouth openings, survival time ↑	Tang et al. (2011)
	Volatile oil	SD rat/*In vivo*	0.1, 0.2, 0.4, 0.8, 1.6 g/kg/6 days	0.1 g/kg	NA	0.25% Tween-80	Degree of pathological damage, hemispheric edema rate, neurological function score, cerebral infarction volume ratio, FIB content ↓	Zhou et al. (2015)
	Volatile oil	Wistar rats/*In vivo*	0.4 g/kg/NA	0.4 g/kg	NA	0.25% polysorbate aqueous solution	Rat tail bleeding time, PT, APTT ↑; neurobehavioral score, cerebral infarction rate, FIB content ↓	Li et al. (2020)
	Volatile oil	Rat cortical nerve cells/*In vitro*	10, 20, 40 μg/ml/24 h	10 μg/ml	NA	Neurobasal-A medium	Nerve cell proliferation ↑; cell apoptosis, oxidative stress, TLR9 expression ↓	Chen et al. (2020)
	Volatile oil	SD rats/*In vivo*	2 mg/kg/7 d	2 mg/kg	NA	Saline	GSH-PX content, Bcl-2 expression, Bcl-2/Bax ↑; brain water content, MDA content, ET-1 content, Bax protein expression ↓	Huang et al. (2018)
	Whole medicine	SD rats/*In vivo*	1.332 g/kg/3 days	1.332 g/kg	NA	0.2% CMC +5% Tween	SOD activity ↑; brain water content, MDA and TNF-α content, neurological signs and behavioral scores ↓	Ni et al. (2010)
	Whole medicine	SD rats/*In vivo*	1.332 g/kg/3 days	1.332 g/kg	NA	0.2% CMC +5% Tween	Temperature and NO content at 4 h after reperfusion ↓	Ni et al. (2011a)
	Volatile oil	Mouse brain microvascular endothelial cells/*In vitro*	10, 50, 100, 200 μg/ml/24 h	10 μg/ml	NA	DMEM medium	Cell viability ↑; LDH, TNF-α, ICAM-1 content ↓	Zhao et al. (2016)
	Volatile oil	Astrocytes/*In vitro*	0.1, 1, 10 μg/ml/20 h	0.1 μg/ml	NA	High glucose DMEM/F12	IL-6, IL-1, TNF-α, LDH, iNOS, NO, ROS, NF-κB expressions↓	Zhang et al. (2018b)
	Volatile oil	Wistar rats/*In vivo*	0.2, 0.4 g/kg/28 days	0.2 g/kg	NA	NA	CD31, SYP ↑; lesion volume, TNF-α, IL-1β, iNOS, ET-1, NF-κB/p65 positive cell number, activated microglia/macrophages and astrocytes ↓	Zhou et al. (2021)
	Whole medicine	SD rats/*In vivo*	0.667 g/kg/3 days	0.667 g/kg	Nimodipine	5% Tween	VEGF expression ↑; brain water content, cerebral infarction rate, TNF-α content ↓	Wen et al. (2017)
	Volatile oil	Wistar rats/*In vivo*	0.4 g/kg	0.4 g/kg	NA	0.25% polysorbate aqueous solution	BBB permeability during acute cerebral ischemia ↑	Li et al. (2020)
	*Et2O*; 1-Butanol; PET; Water; Whole medicine	KM mice/*In vivo*	1.332 g/kg/3 days	1.332 g/kg	NA	5% Tween	Open BBB, EB content in mice brain ↑	Ni et al. (2011b)
	*Et2O*; 1-Butanol; PET; Water; Whole medicine	KM mice/*In vivo*	1.332 g/kg/3 days	1.332 g/kg	NA	5% Tween	Close the BBB, the EB content in mice brain ↓	Ni et al. (2011c)
	Whole medicine	SD rats/*In vivo*	1.332 g/kg/3 days	1.332 g/kg	NA	5% Tween	Improve the BBB ultrastructure of the frontal and parietal cortex on the ischemic side	Ni et al. (2011d)
	Whole medicine	ICR mice, SD rats/*In vivo*	0.3 g/kg/7 days	0.3 g/kg	NA	0.1% CMC-Na	EB distribution in cortex, hippocampus and hypothalamus, Rh123 distribution in hippocampus and striatum, hippocampal permeability index Kp ↑; does not affect the ultrastructure of BBB	Ding et al. (2015)
Sedative and anticonvulsant	EtOH	ICR mice/*In vivo*	25, 50, 100, 200 mg/kg (i.g.); 12.5, 25, 50 mg/kg (Nasal administration)	25 mg/kg (i.g.); 12.5 mg/kg (Nasal administration)	Diazepam	3% Tween-80	Sleep time induced by pentobarbital ↑; epilepsy and mortality induced by pentylenetetrazol PTZ ↓	Guo et al. (2011)
	Volatile oil	KM mice/*In vivo*	0.4323 g/kg/7 days	0.4323 g/kg	NA	Saline	Convulsion incubation period ↑; the number of spontaneous activities in mice, the number of convulsions caused by bitter toxin or strychnine↓	Huang et al. (2020a)
Excitement center	Volatile oil	KM mice/*In vivo*	0.4323 g/kg/7 days	0.4323 g/kg	NA	Saline	Pentobarbital sodium sleep duration ↓	Huang et al. (2020a)
	Volatile oil	Mice/*In vivo*	0.9 g/kg/7 days	0.9 g/kg	NA	Saline	Aspartic acid and reduced glycine ↑	Zhang et al. (2012a)

APTT, activated partial thromboplastin time; BBB, blood-brain barrier; EB, evans blue; Et2O, ethyl ether; FIB, fibrinogen; GSH-Px, glutathione-Px; ICAM, intercellular cell adhesion molecule; IL, interleukin; iNOS, inductible nitric oxide synthase; KM, kunming; LDH, lactate dehydrogenase; MDA, malondialdehyde; NA, not available; NF-κB, nuclear factor kappa-B; NO, nitric oxide; PET, petroleum ether; PT, prothromboplastin time; ROS, reactive oxygen species; SD, sprague-dawley; SOD, superoxide dismutase; SYP, synaptophysin; TLR, toll-like receptor; TNF, tumor necrosis factor.

**TABLE 4:** Pharmacological effects of storax on cardiovascular diseases (”↓,” decrease; “↑,” increase).

Pharmacological activities	Extract	Model/Method	Dose/Duration	Minimal active dose	Control		Results	References
					Positive	Negative		
Anti-myocardial ischemia	Whole medicine	SD rats/*In vivo*	0.167, 0.333, 0.667 g/kg/3 days	0.167 g/kg	Nitroglycerin	5% Tween	LVAP, LVEDP, LVSP, *LVDP*, myocardial infarction rate, CK-MB, LDH, AST levels ↓	Chen et al. (2019)
	Whole medicine	SD rats/*In vivo*	200, 400, 800 mg/kg/14 days	400 mg/kg	Diltiazem	Krebs-Henseleit	Bcl-2 expression ↑; LDH, CK, Bax expressions, cardiomyocytes apoptosis, myocardial tissue damage ↓	Wang and Gou, (2019)
	Volatile oil	SD rats/*In vivo*	0.2, 0.4, 0.8 g/kg/21 days	0.4 g/kg	NA	0.25% Polysorbate-80	Myocardial infarction area, WBV, PV, CK-MB, LDH expressions↓	Mu et al. (2019)
	Whole medicine	Bovine adrenal medulla cells/*In vitro*	0.5, 5, 50 mg/L/NA	0.5 mg/L	NA	KRP buffer	CA secretion caused by ACh and Ver ↓	Liu et al. (2010b)
	Et2O; 1-Butanol; PET; Water; Whole medicine	Mice/*In vivo*	1.332 g/kg/3 days	1.332 g/kg	NA	5% Tween +0.2% CMC-Na	Survival time ↑; oxygen consumption ↓	Xu et al. (2010)
	Volatile oil	Mice/*In vivo*	600, 800 mg/kg/1 day	600 mg/kg	Propranolol	1% Tween-80	Hypoxia tolerance time ↑	Wang et al. (2013a)
	Volatile oil	Guinea pigs/*Ex vivo*	50, 100 ng/ml/1 day	50 ng/ml	Verapamil hydrochloride	0.1% ethanol	Coronary flow, cardiac diastolic velocity ↑	Wang et al. (2013a)
	Volatile oil	LDL suspensions/*In vitro*	2.5 mg/1, 3, 6 h	2.5 mg	NA	NA	LDL oxidation levels ↓	Andrikopoulos et al. (2003)
	Whole medicine	SD rats/*In vivo*	2, 4 mg/kg/5 days	2 mg/kg	NA	0.1% CMC-Na	Thrombosis, blood viscosity erythrocyte pressure product, platelet aggregation rate ↓	Huang et al. (2019)
	EtOH	SD rats/*In vivo*	100, 200 mg/kg/7 days	200 mg/kg	NA	0.5% CMC-Na	Thrombus of length, wet and dry weight↓	Wang et al. (2013a)
	EtOH	Rabbits/*In vivo, In vitro*	100 mg/kg/1, 4, 24 h (*In vivo*); 0.5, 1 mg/ml/NA (*In vitro*)	100 mg/kg (*In vivo*); 0.5 mg/ml (*In vitro*)	NA	0.1% CMC-Na	Intraplatelet cAMP content, PRT, PT, KPTT, fibrinolytic enzyme activity↑; plasma FIB content ↓	Zhang et al. (2012b)
	Whole medicine	Rabbits, SD rats/*In vivo*	1.2 mg/ml/NA	1.2 mg/ml	Aspirin	Sodium Phosphate Buffer	Platelet aggregation ↓	Gu et al. (2012)
	EtOH	SD rats/*In vivo*	2, 4 mg/kg/NA	2 mg/kg	NA	0.1% CMC-Na	Platelet aggregation rate ↓	Gu et al. (2012)
Anti-arrhythmia	EtOH	Mice/*In vivo*	200, 400 mg/kg/1 day	200 mg/kg	Mexiletine Hydrochloride	0.5% Tween-80	Time of arrhythmia occurrence, number of arrhythmia occurrence per unit time ↓	Wang et al. (2013a)
	NA	HEK293T/*In vitro*	5%/NA	5%	NA	NA	Kir 2.1 Inward and outward currents ↓	Ren et al. (2017b)

AST, aspartate aminotransferase; CA, catecholamine; CK-MB, creatine kinase isoenzymes; Et2O, ethyl ether; EtOH, ethanol; FIB, fibrinogen; KPTT, kaolin partial thrombin time; LDH, lactate dehydrogenase; LVAP, left ventricular pressure; LVDP, left ventricular diastolic pressure; LVEDP, left ventricular end-diastolic pressure; LVSP, left ventricular systolic pressure; NA, not available; PET, petroleum ether; PRT, plasma recalcification time; PT, prothromboplastin time; PV, plasma viscosity; SD, sprague-dawley; WBV, whole blood viscosity.

### 5.1 The Effects on Cerebrovascular Diseases

ST has a variety of cerebral protective effects, for instance, anti-cerebral ischemia, anti-convulsion and central excitement, as demonstrated in Table 3.

#### 5.1.1 Anti-cerebral Ischemia

**FIGURE 5:**
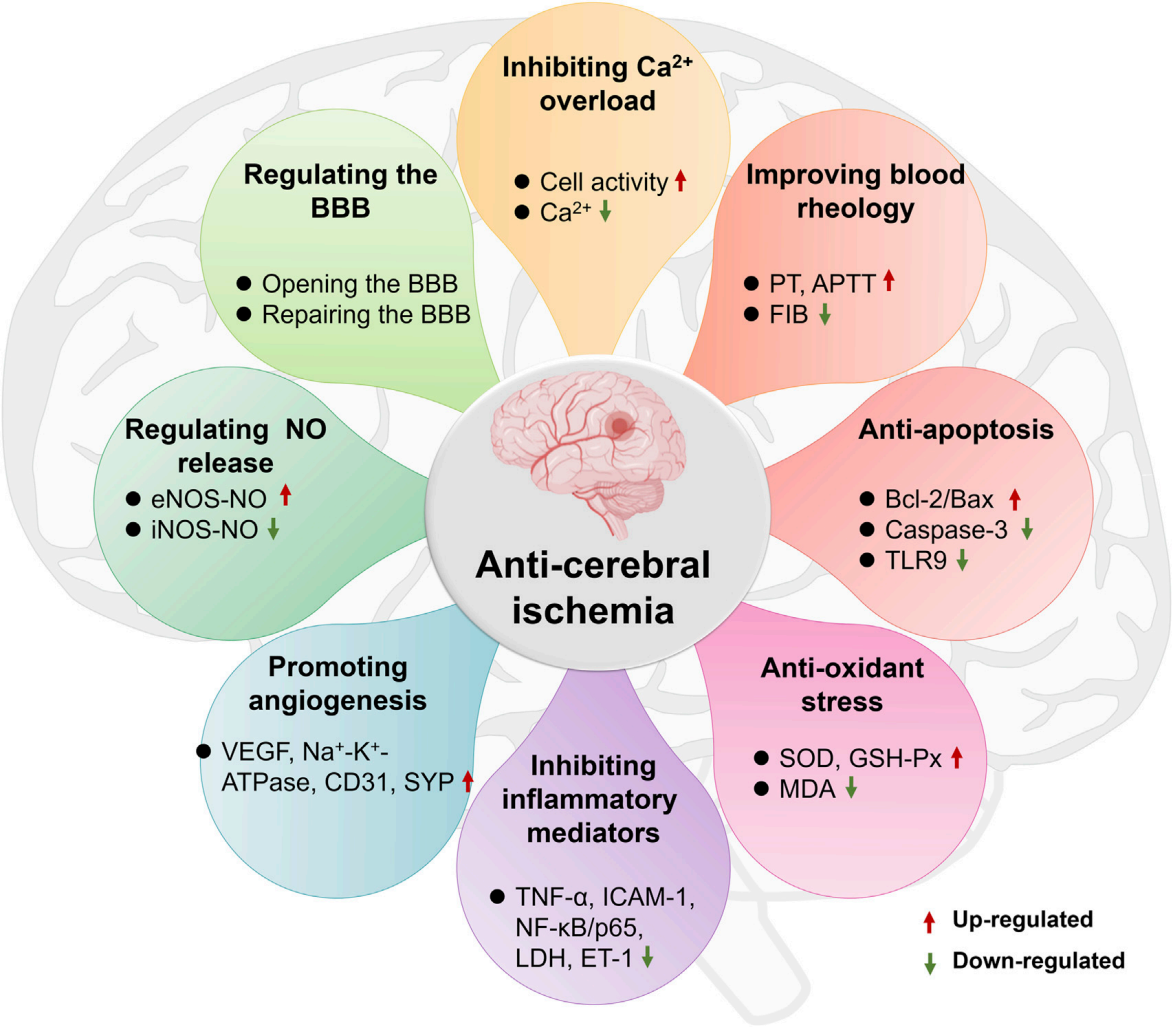
The main pharmacological mechanism of storax on cerebral ischemia.

Ischemic brain injury is a complex pathophysiological process involving multiple factors such as NO neurotoxicity, inflammatory mediator stimulation and release, calcium overload and apoptosis. However, ST has shown the characteristics of multi-link and multi-pathway effects in the intervention of cerebral ischemic pathological damage. It was showed that ST significantly inhibited Na_2_S_2_O_4_-induced ischemic-hypoxic injury in PC12 cells as well as OGD/R-induced injury in cerebral microvascular endothelial cells, astrocytes and cortical neuronal cells, and enhanced cell viability (Liu et al., 2010a; Wang D. et al., 2012). It was also found that ST significantly increased the number of mouth openings in mice after decapitation and also prolonged the duration of mouth opening in mice injected with saturated magnesium chloride solution causing hypoxia (Tang et al., 2011), as well as reducing the degree of histopathological damage to the brain and the volume of cerebral infarction in MCAO rats. These results have indicated that ST has a noticeable improvement on ischemic brain tissue, and its primary mechanisms of action are shown in Figure 5, including inhibition of Ca^2+^ overload, improvement of blood rheology, anti-apoptosis of brain cells, anti-oxidative stress, inhibition of inflammatory mediators, promotion of angiogenesis, regulation of NO production and the blood-brain barrier (BBB). The main mechanisms as follows:

① Inhibiting Ca^2+^ overload

Under normal conditions, cells are in a dynamic equilibrium of calcium homeostasis, which is important for the maintenance of cellular physiological functions. Ca^2+^ is the most important “second messenger”, which plays a crucial role in the physiological activities of cells and the structural and functional integrity of neurons. Once the homeostasis of Ca^2+^ concentration in neurons is maladjusted, calcium overload occurs, which will lead to degeneration and death of nerve cells (He et al., 2020). While ST can reduce the excessive influx of Ca^2+^ in PC12 cells damaged by ischemia and hypoxia to improve cell viability (Liu et al., 2010a; Wang D. et al., 2012).

② Improving blood rheology

ST has been found to alter blood viscosity after cerebral ischemia. ST can significantly prolong plasma prothromboplastin time (PT), activated partial thromboplastin time (APTT), and reduce fibrinogen (FIB) content after cerebral ischemia. These explained that the anti-ischemic effect of ST may be related to improved cerebral blood flow by improving post-ischemic blood hypercoagulation (Zhou et al., 2015; Li et al., 2020).

③ Anti-apoptosis of brain cells

ST is able to inhibit the apoptosis of cortical nerve cells injured by CI/RI and reduce the expression of Caspase-3 protein significantly, whose regulatory mechanism is related to TLR9 (Chen, 2020). It may up-regulate the Bcl-2/Bax ratio in ischemic brain tissues of CI/RI rats as well (Huang et al., 2018). What’s more, it can ameliorate the damage of capillary endothelial cells, astrocytes and neurons in the frontal-parietal cortex of the ischemic hemisphere in rats to varying degrees (Ni et al., 2010; Ni et al., 2011a). These outcomes have evidenced that ST has an anti-apoptosis impact on brain cells.

④ Anti-oxidant stress

During cerebral ischemia, the imbalance between free radical generation and removal causes free radicals to accumulate, destroys the protein components in the membrane structure, causes membrane lipid peroxidation and leads to membrane damage, mitochondrial dysfunction, lysosome rupture, cell lysis, and tissue edema such a series of damage effects. These further promote the formation of oxidative stress, which in turn aggravates brain tissue damage (Liu et al., 2015). Malondialdehyde (MDA) is a breakdown product of lipid peroxidation and is one of the indicators reflecting the level of tissue oxidation. Superoxide dismutase (SOD) and glutathione peroxidase (GSH-Px), which are the main antioxidant enzymes in the body, are free radical scavengers. Insufficient activity of SOD and GSH-Px means free radical scavenging ability decline. ST can increase the SOD activity of cortical nerve cells injured by CI/RI, reduce the level of MDA, and increase the level of GSH-Px in the serum of CI/RI rats (Huang et al., 2018; Chen, 2020). These results have testified that ST has an antioxidant effect.

⑤ Inhibiting the release of inflammatory mediators

After cerebral ischemia, the expression of a large number of inflammatory factors is upregulated, which promotes neutrophils to adhere to the vascular wall and enter the central nervous tissue. Subsequently, monocytes and macrophages infiltrate to destroy the endothelial cells and basement membrane of cerebral microvessels, further leading to vasogenic edema and bleeding. ST has been found to ameliorate OGD/R-induced damage to brain microvascular endothelial cells, astrocytes and cortical neuronal cells, increase cell viability, decrease LDH, TNF-α and ICAM-1 release, and reduce NF-κB expression (Zhao et al., 2016; Zhang M. et al., 2018). It was also found that ST significantly suppressed TNF-α and IL-1β expression in brain tissue and ET-1 levels in serum, reduced the number of NF-κB/p65-positive cells, and decreased the number of activated microglia/macrophages and astrocytes while reversing their morphological change 24 h after cerebral ischemia in rats (Zhou et al., 2021), and also significantly reduced hemispheric edema rates, neurological function scores and brain infarct volume ratios after cerebral ischemia in rats. These results have confirmed that ST may exert neuroprotective effects by inhibiting the NF-κB signaling pathway and thus reducing the expression of related inflammatory factors.

⑥ Promoting angiogenesis

Angiogenesis after cerebral ischemia is essential to promote the recovery of nerve function. VEGF is a multifunctional factor that acts on vascular endothelial cells and is a specific mitogenic source of endothelial cells. It can promote endothelial cell division, proliferation and metastasis. VEGF can induce the proliferation of endothelial cells by activating the expression of its receptors, thereby promoting the formation of new blood vessels and the establishment of collateral circulation, which is currently recognized as the most critical factor in angiogenesis (Wang H. et al., 2020). ST can increase VEGF content in serum and Na^+^-K^+^-ATPase activity in ischemic brain tissues of CI/RI rats (Wen et al., 2017). Long-term neurological studies have found that ST significantly alleviates neurological impairment starting 7 days after cerebral ischemia and continuing up to 28 days, with a significant reduction in lesion volume 28 days after stroke, as well as increasing the density of CD31 and SYP around the infarcted area (Zhou et al., 2021).

⑦ Regulating the production of NO

NO is believed to perform at least four actions in the central nervous system (CNS): 1) As a vasodilator, it plays an important role in controlling cerebral blood flow. 2) As a neuroprotective agent, it can reduce excessive calcium influx. 3) As a neurotransmitter, it can increase cAMP level by combining with guanylate cyclase and inactivate toxic oxygen-free radicals. But at high concentrations, it can directly damage DNA and interfere with the respiratory chain. 4) As a cytotoxin, it may contribute to neuronal cell death (Gao et al., 2020; Lv et al., 2020). Whereas NOS is a key determinant of the dual role of NO, different subtypes of NOS have different roles in cerebral ischemic injury. NO production by nNOS and iNOS is associated with nerve injury. nNOS is expressed in the early stages of ischemic neuronal injury, with activity rising 10 min after ischemia and peaking at 3 h, whereas iNOS is expressed in the late stages of ischemic neuronal injury, with activity rising 12 h after ischemia and peaking at 48 h (Liu et al., 2020). In contrast, NO produced by eNOS has a neuroprotective effect, dilating cerebral blood vessels and increasing cerebral blood flow in ischemic areas, with activity rising at 1 h and peaking at 24 h (Yagita et al., 2013). Studies have found that ST can not only increase the expression of eNOS and stimulate the production of NO to play a neuroprotective effect (Liu et al., 2010a), but also inhibit the production of iNOS and eliminate the neurotoxicity of NO to play a dual brain protection effect (Ni et al., 2011a; Zhang M. et al., 2018).

⑧ Regulating the BBB

The BBB is a protective structure that exists between blood and brain tissue. It is responsible for regulating the exchange of substances between blood and brain tissue and allowing the nutrients needed by brain tissue in blood circulation (Huang Y. et al., 2020). The passage of substances can also effectively prevent the intrusion of harmful substances, thereby maintaining the stability of the internal environment of the CNS (Jiang et al., 2018). In pathological conditions, let’s say ischemic stroke could make the BBB disrupted, with subsequent extravasation of blood components into the brain, impairing normal neuronal function. So how to enhance BBB repair is essential to promote long-term functional recovery after ischemic stroke. Under physiological conditions, ST significantly increased the level of Evans Blue (EB) in mice brain tissue and had an opening effect on the BBB in normal mice, which increased with time and was significantly different from the control group at 72 h, and the results of all studies are consistent (Ni et al., 2011b; Li et al., 2020).

In the state of cerebral ischemia, Caixia Ni et al. considered that ST tends to reduce the content of EB in brain tissue (Ni et al., 2011c), which seems to be able to repair BBB and prevent harmful substances from entering the brain parenchyma. In contrast, Dongna Li et al. believed that ST could increase the openness of the BBB in the acute phase of cerebral ischemia, that is, 2 h of ischemia. By 48 h, there was a significant increase in Evans Blue transmission compared to the model group (Li et al., 2020). Similar to the results of Li et al., Liu et al. observed that ST could significantly increase the concentration of combined sulpiride in the brain and blood of cerebral ischemia rats by using microdialysis technology (MD) combined with high-performance liquid chromatography (HPLC), indicating that ST could increase the permeability of BBB and promote the penetration of sulpiride through BBB (Liu et al., 2012). These two findings seem to be somewhat contradictory, but at the same time they are consistent with the characteristics of aromatic resuscitation Chinese medicines, which on the one hand can exert neuroprotective effects, and on the other hand can open the BBB so that the drug itself, as well as the concomitant drugs, can enter the brain parenchyma and exert neuroprotective effects more directly, while the exact time window when the BBB is opened still needs to be further examined.

The addition of benzaldehyde and vanillin, the active ingredients of ST, to caco-2 cells with high expression of P-glycoprotein (P-gp), was found to significantly promote the uptake of alkaline rhodopsin-123 (Rho-123) by caco-2 cells, suggesting that the inhibition of P-gp efflux function may be one of the mechanisms by which ST improves the permeability of the BBB (Yang et al., 2012). Further research found that ST significantly increased the distribution of EB in the cortex, hippocampus and hypothalamus of normal rats, as well as the distribution of Rho-123 in the hippocampus and striatum, and increased the permeability index Kp of the hippocampus, suggesting that ST could induce the opening of the BBB in the cortex, hippocampus and hypothalamus. It is speculated that the opening effect of ST on the hippocampus is related to the inhibition of P-gp function, while the increase of EB distribution in other brain regions may have nothing to do with P-gp function and may be related to other ATP-binding box proteins (such as Mrps and Bcrp) (Ding et al., 2015). In addition, ST has been found not to affect the ultrastructure of the BBB in normal rats (Ding et al., 2015), but to improve the cortical BBB ultrastructure in the frontoparietal region of the ischemic side of the hemisphere in CI/RI rats (Ni et al., 2011d). In summary, ST can not only open the BBB non-destructively, but also directly repair the BBB. Still, its time window and mechanism of action need to be further clarified.

#### 5.1.2 Sedation and Anticonvulsant

Convulsion is a symptom of central nervous overexcitation caused by various causes. Pentatetrazole induces convulsion by blocking the activity of γ -aminobutyric acid (GABA) in the CNS. Studies have found that ST can significantly prolong the incubation period of pentatetrazole-induced convulsion and thus reduce the mortality rate of convulsion. These results suggest that ST may play an anticonvulsant effect through the GABA neuroregulatory pathway (Guo et al., 2011). It also has significant effects on convulsion induced by strychnine and picrotoxin (Huang et al., 2020a). It was also found that long-term inhalation of volatile oils of ST could prolong pentobarbital induced sleep, and nasal inhalation had a sedative and anticonvulsant effect faster than gavage, and significantly reduced the content of MDA and glutamate in brain tissue, suggesting that volatile oil of ST had a certain sedative and antioxidation effect. As the excitatory amino acids are released in the state of oxidative stress, it is believed that the anticonvulsant effect of ST is also related to its antioxidant effect, which provides a theoretical basis for its clinical use in the treatment of convulsions (Guo et al., 2011). Nasal administration itself can improve drug dosage because there are two ways for drugs to be absorbed into the brain through the nose: one is directly absorbed into the brain through the olfactory mucosa; the other is absorbed into the systemic circulation through the mucosa of the respiratory area, and then transported into the brain through BBB, which is effective due to rapid absorption (Zhao et al., 2012; Pardeshi and Belgamwar 2013; Long et al., 2020). Therefore, nasal administration is very important for the treatment of acute central system diseases, and ST naturally adjusts the advantages of BBB combined with the optimization of drug delivery mode is of great significance for the development of new and efficient central targeted drugs.

#### 5.1.3 Excitatory Centers

Aromatic resuscitation drugs have a two-way regulating effect on the excitability of the CNS, which is both “tranquilizing the mind” and “inducing-resuscitation.” Studies have shown that oral administration of ST can increase the content of aspartic acid in the striatum of rats and decrease the content of glycine, which plays an excitatory role in neurotransmitters. Different from the previous results of prolonging the sleep duration of pentobarbital sodium, ST can also shorten the sleep duration of pentobarbital sodium, showing the effect of brain awakening and excitation (Zhang N. et al., 2012; Huang et al., 2020a). The reason for the two opposite results may be that prolonged inhalation of the volatile oil of ST is equivalent to an increased dose of the drug, which has a sedative effect similar to that of “gaseous anesthesia,” while oral administration has an “inducing-resuscitation” effect, which plays a role of excitement center. However, this hypothesis has not been confirmed in the same overall experiment, so it should be verified by means of multiple models and multiple indicators.

### 5.2 The Effects on Cardiovascular Diseases

ST mainly treats cardiovascular diseases in terms of anti-myocardial ischemia and anti-arrhythmia, as described in Table 4.

#### 5.2.1 Anti-myocardial Ischemia

**FIGURE 6:**
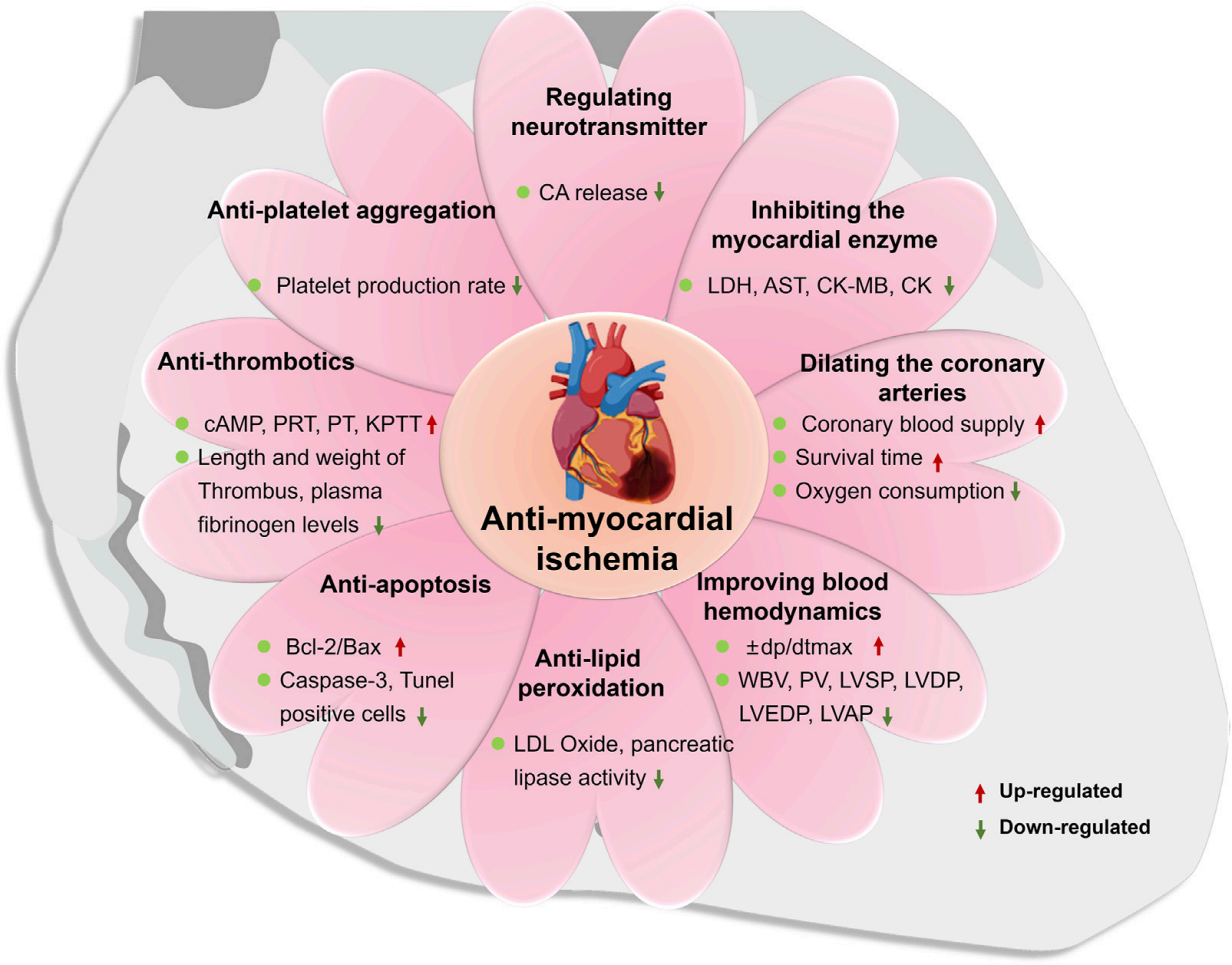
The main pharmacological mechanism of storax on myocardial ischemia.

Myocardial ischemia is a pathological condition in which the blood supply and perfusion of the heart are reduced and the oxygen supply is reduced, which leads to abnormal energy metabolism and the heart cannot work normally. It is the main pathogenesis of a variety of cardiovascular diseases, such as myocardial infarction and atherosclerosis. A number of studies have confirmed that ST has an inhibitory effect on the myocardial infarction area and myocardial infarction rate in rats with myocardial ischemia. Pathological examination shows that it can improve myocardial cell necrosis, degeneration, bleeding and edema caused by ischemia, indicating that it has a protective effect on the ischemic myocardium (Chen et al., 2019; Mu et al., 2019; Wang and Gou, 2019). Due to the complex and diverse pathogenesis of myocardial ischemia, the main mechanisms of ST in anti-myocardial ischemia are shown in Figure 6, including regulation of neurotransmitters, regulation of myocardial enzyme spectrum, dilation coronary arteries, improvement of hemorheology and hemodynamics, anti-lipid quality peroxidation, anti-cardiomyocyte apoptosis, anti-thrombotics and anti-platelet aggregation. The main mechanisms as follows:

① Regulating the release of neurotransmitters

About 3/4 of ischemic heart diseases are caused by autonomic nerve dysfunction, which is typically characterized by a sharp decrease in vagal tone accompanied by an increase in sympathetic nerve activity (Zhang D. et al., 2018). Increased sympathetic excitability leads to increased catecholamine (CA) neurotransmitter secretion. CA is an important physiological substance that regulates cardiovascular activities. Under normal conditions, it acts on adrenergic receptors and dopamine receptors to produce contraction of blood vessels, enhancing myocardial contractility and improving the excitability of cardiomyocytes. It is an important neurohumoral hormone that maintains the physiological functions of the body, and its increase has a certain rhythm. However, in the pathological state, this rhythm is disrupted, which is manifested by the continuous or sudden increase of catecholamine, and the excessive increase of catecholamine has direct damage to the myocardium. The release of large amounts of catecholamines can cause an increase in myocardial oxygen consumption, resulting in coronary artery spasm, leading to myocardial ischemia and hypoxia, and even necrosis. However, ST can inhibit the secretion of CA caused by acetylcholine stimulation of bovine adrenal medulla cells in a dose-dependent manner. The mechanism is mainly related to the nAChR pathway on the cell membrane and the voltage-dependent sodium ion channel (Liu et al., 2010b), suggesting that ST can achieve the effect of “brain regulates the heart” by regulating the CNS in the treatment of cardiovascular diseases.

② Inhibiting the myocardial enzyme profile

Cardiac enzymes are enzymes within cardiac muscle cells that catalyze cardiomyocyte metabolism and regulate cardiac electrical activity in cardiac muscle cells. In clinical practice, serum glutamic-oxalacetic transaminase (AST), lactate dehydrogenase (LDH), creatine kinase (CK) and creatine kinase isoenzyme (CK-MB) are commonly used as reference indexes for myocardial ischemia and hypoxia injury. Once the cardiomyocytes become necrotic and ruptured, these enzymes will be released into the serum, causing the enzyme concentration to increase. Several studies have demonstrated that both pre-administration and combined prophylactic and therapeutic administration of ST inhibited the rise in serum cardiac enzymes induced by myocardial ischemia (Chen et al., 2019; Mu et al., 2019; Wang and Gou, 2019), suggesting that ST has a role in modulating the cardiac enzyme profile in rats with myocardial ischemia.

③ Dilating the coronary arteries

The imbalance between myocardial oxygen demand and supply can lead to myocardial ischemia and hypoxia, causing metabolites such as lactic acid to accumulate in the heart tissue, resulting in an attack of angina. Dilating the coronary arteries reduces myocardial oxygen consumption and improves coronary blood supply. Placing mice pre-administered with ST in a hypoxic environment prolongs survival time by reducing oxygen consumption (Xu et al., 2010; Wang Y. et al., 2013). It has also been shown that ST increases coronary blood flow as well as cardiac diastolic velocity in guinea pigs with ligated lateral left ventricular vessels (Wang Y. et al., 2013). The results of these studies all tentatively suggest that ST has a coronary vasodilating effect, but its mechanism still needs to be studied in depth.

④ Improving blood rheology and hemodynamics

Blood rheology and hemodynamics focus on the viscosity of blood and its movement through the circulatory system. High blood viscosity, poor fluidity, the risk of myocardial infarction is higher, on the contrary, low blood viscosity, good fluidity, the risk of myocardial infarction is small. It was found that ST significantly reduced whole blood viscosity (WBV) and plasma viscosity (PV) in rats with myocardial infarction. In addition, ST inhibited increases in left ventricular systolic pressure (LVSP), left ventricular diastolic pressure (LVDP), left ventricular end-diastolic pressure (LVEDP) and mean left ventricular intraventricular pressure (LVAP) in rats with myocardial ischemia (Chen et al., 2019), and also increased the reduction of ±dp/dtmax induced by ischemia, and acted earlier than diltiazem (Wang and Gou, 2019). The above research results indicate that ST can reduce blood viscosity and improve its fluidity, thereby improving left ventricular function.

⑤ Anti-lipid peroxidation

Low-density lipoprotein (LDL) is the main carrier of cholesterol for transport to peripheral tissue cells and is associated with the deposition of cholesterol into endothelial tissue, which can lead to the formation of atheromatous plaques, while the rupture of vulnerable atherosclerotic plaque is a major cause of myocardial infarction (Gustafsen et al., 2017). Moreover, LDL is highly susceptible to oxidation in an atherogenic environment. Oxidized low-density lipoprotein has been shown to enhance the formation of foam cells in atherosclerotic plaques, thereby increasing the risk of coronary heart disease, such a vicious circle (Stein et al., 2010). ST can protect low-density lipoprotein from oxidation and prevent coronary heart disease, and its active components may be pentacyclic triterpenoids (Andrikopoulos et al., 2003). Inhibition of pancreatic lipase activity is one of the most effective methods for the treatment of obesity and obesity-related metabolic disorders, such as atherosclerosis and hyperlipidemia. ST contains five pentacyclic triterpenoid acids, which have a strong inhibitory effect on pancreatic lipase, with IC50 values ranging from 0.49 to 6.35 μm. Among them, oleanonic acid and betulonic acid are the most effective two pancreatic lipase inhibitors, and they have a strong inhibitory effect on pancreatic lipase in a reversible and mixed manner, revealing the chemical composition of ST for the treatment of lipid metabolic diseases such as coronary heart disease (Wang et al., 2020b).

⑥ Anti-apoptosis of cardiomyocytes

Apoptosis, also known as programmed cell death, is an active and orderly cell death mode controlled by genes (Zhai et al., 2017). Myocardial apoptosis occurs during ischemia and hypoxia. Therefore, inhibition of myocardial apoptosis has become a research direction in the treatment of myocardial ischemia. The molecular mechanisms of apoptosis are complex. The Bcl-2 family, calpain and caspases are all involved in the initiation, occurrence, and development of apoptosis. Apoptosis-promoting protein Bax and apoptosis-inhibiting protein Bcl-2 restrict each other, and their ratio reflects the state of apoptosis. Studies have found that ST can significantly reduce the ratio of Bax to Bcl-2, suggesting that it can inhibit cardiomyocyte apoptosis. Further Tunel staining analysis revealed that ST significantly reduced the number of positive cardiomyocytes and its regulatory mechanism may be related to the PI3K/Akt signaling pathway, suggesting that it may improve myocardial ischemia and myocardial ischemia/reperfusion (MI/RI) injury in rats by inhibiting myocardial apoptosis (Chen et al., 2019; Wang and Gou, 2019).

⑦ Anti-thrombotics

The occurrence and development of thrombotic diseases are not only related to vascular wall lesions and blood flow disorders but also related to platelet function, coagulation function and fibrinolytic activity. *In vitro* experiments on rat thrombosis showed that ST could significantly inhibit thrombus length, wet weight and dry weight of thrombus (Wang Y. et al., 2013), and *in vivo* experiments showed that ST could inhibit thrombus formation, reduce blood viscosity and hematocrit in rats (Huang et al., 2019). *In vivo* and *in vitro* experiments in rabbits have also shown that ST can significantly increase the cAMP content in platelets, prolong plasma recalcification time (PRT), prothrombin time (PT) and kaolin partial thrombin time (KPTT), reduce plasma FIB content and plasmin activity (Zhang N. et al., 2012). These results all tentatively suggested that ST could alleviate blood coagulation and act as an antithrombotic agent. Neverthless, there are few relevant studies and animal models such as rabbits and rats are costly, difficult to manipulate and prone to thrombolysis. Future studies can use ferric chloride to induce thrombosis in zebrafish models, which is much more microdosing and efficient than other traditional animal thrombosis models and can screen antithrombotic active ingredients in high throughput.

⑧ Anti-platelet aggregation

Platelet plays an important role in thrombosis and inflammatory response, and its role runs through the whole process of cardiovascular diseases. Its activation and aggregation are related to unstable atherosclerotic lesions and are serious risk factors for patients with coronary heart disease (Moraes, et al., 2013). ST and its component cinnamic acid have obvious anti-aggregation effects on rabbit and rat platelets. *In vitro* experiments showed that ST and cinnamic acid used collagen as the inducer, the inhibition rates for rabbits were 33 and 52%, and the inhibition rates for rats were 24 and 42%. With ADP as an inducer, the inhibitory rates for rabbits were 32 and 72%, and for rats were 35 and 77%. The effect of cinnamic acid was equivalent to that of aspirin and ferulic acid (Gu et al., 2012). These results indicated that ST had an inhibitory effect on platelet aggregation. Unfortunately, only the cinnamic acid contained in it has been tested, and it is impossible to judge whether other ingredients have a more significant inhibitory effect, which should be discussed in the future.

Although the above studies reveal the pharmacological mechanism of ST on myocardial ischemia and hypoxia from lots of aspects, it is still necessary to supplement the longitudinal and in-depth molecular mechanism.

#### 5.2.2 Anti-arrhythmia

Arrhythmia refers to the abnormality of cardiac rhythm, frequency, or activation sequence caused by cardiac pacing and conduction dysfunction, mainly manifested as tachycardia, bradycardia, arrhythmia, conduction block and arrest, which can lead to syncope, sudden death, and other critical situations (Bernardi et al., 2020). Modern antiarrhythmic drugs for arrhythmia are effective but have a large number of adverse effects, in addition to liver and kidney damage, and even some antiarrhythmic drugs have the same arrhythmogenic adverse effects (Wang X. et al., 2013). Traditional Chinese medicine has good clinical effects and safety in anti-arrhythmia. It can improve the quality of life of patients while improving clinical symptoms. Studies have shown that ST can significantly reduce the incidence of chloroform-induced arrhythmias in mice, with a tendency to enhance with increasing dose (Wang Y. et al., 2013). Ren et al. screened 25 kinds of traditional Chinese medicines that were thought to alleviate arrhythmia by the TI+flux screening method. It was found that ST could block the inward and outward currents of Kir2.1, and it was the first traditional Chinese medicine to block Kir2.1 and its mutants, which might become a targeted drug for type 3 short QT syndrome (sqt3). Further studies found that the effective component of its role was hydrocinnamic acid (Ren et al., 2017a; Ren et al., 2017b), but its mechanism still needs to be clarified.

## 6 Conclusion and Future Perspectives

**FIGURE 7:**
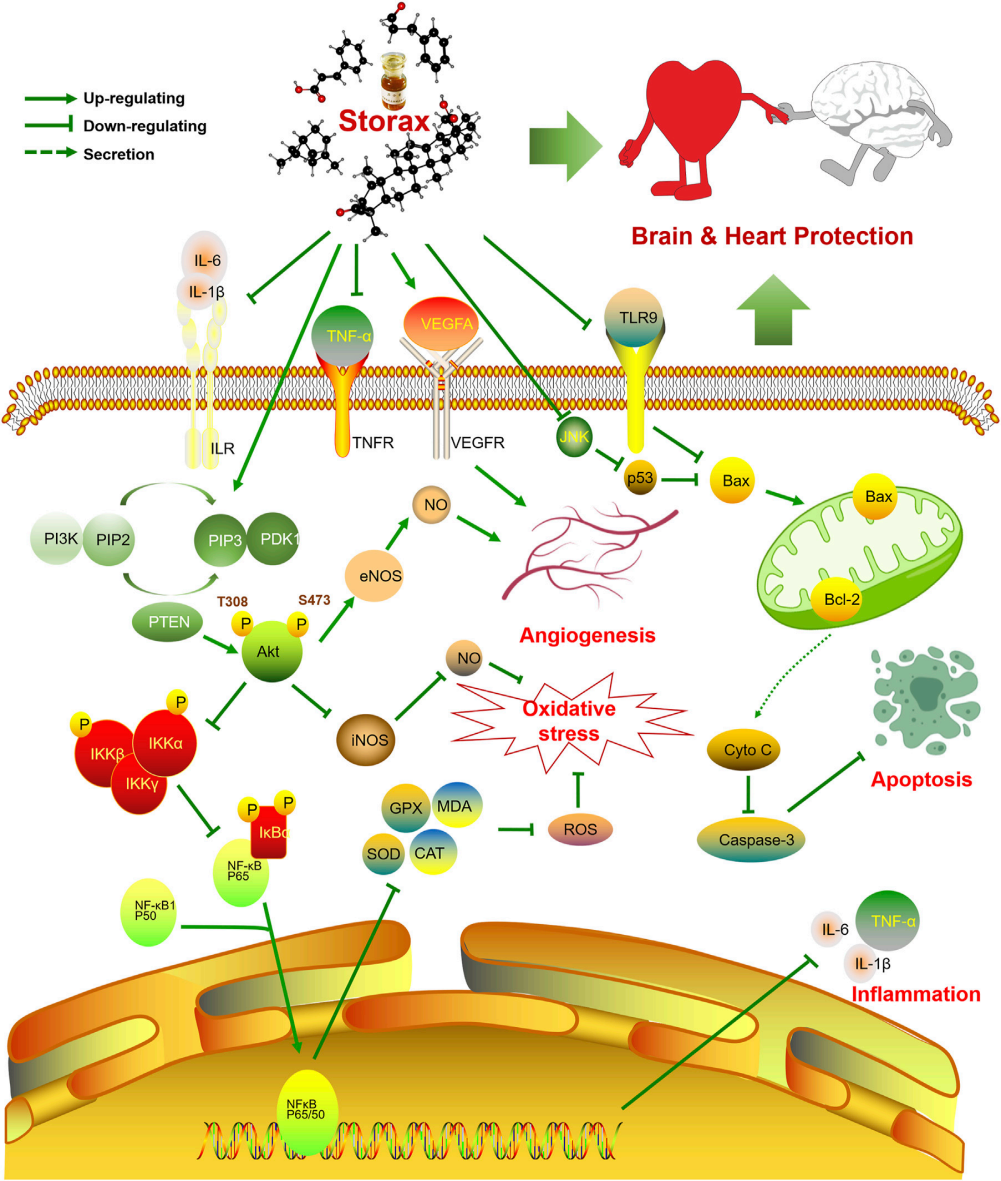
Schematic diagram of the regulatory mechanism of the brain-heart protective effect of storax. Storax can activate PI3K/Akt signaling pathway and inhibit NF-κB signaling pathway, which in turn can play a role in regulating NO production, anti-inflammatory, anti-oxidative stress and pro-angiogenic pharmacological effects, as well as anti-apoptosis by elevating Bcl-2 and inhibiting the expression of Caspase-3 and Bax, thus playing a co-protective role in the brain and heart.

As one of the most popular folk medicines, ST has a history of widespread use in Asia and Africa, with remarkable benefits in the prevention and treatment of cardiovascular and cerebrovascular diseases (e.g., angina pectoris, myocardial infarction, stroke and cerebral atherosclerosis). In conclusion, ST can play a brain-heart protective role through the common pharmacological mechanisms of anti-inflammation, anti-oxidative stress, anti-apoptosis and pro-angiogenesis, the specific regulation schematic is shown in Figure 7. However, the present domestic and international literature does not go far enough into the investigation of ST. Some reports are outdated, with single doses, lack of controls or *in vitro* studies only. The pharmacological research of herbal medicine should be carefully designed, with detailed records of dose, route and frequency of administration, appropriate models and accurate measurement methods. Neither *in vitro* nor in silico can replicate the complex environment of the organism, so it is not enough to accurately evaluate the effects of drugs. Therefore, we recommend the use of a combination of *in vivo* and *in vitro* studies, with multiple doses being investigated simultaneously for pharmacological studies, following the requirements of the consensus document (Heinrich et al., 2020). This review explores future research directions and priorities for ST as indicated below:

① Digging deeper into the chemical composition of ST on the protection for the brain and heart. Although a large number of compounds have been isolated and identified from ST, only a few have been evaluated pharmacologically. Therefore, an in-depth study of the chemical composition of ST and its pharmacological action, especially the mechanism of action of its bioactive components, in order to illustrate the correlation between ethnomedical use and biological activity, will undoubtedly be the focus of further research. As the study of the chemical composition of its potency is still at the stage of crude extraction, its specific active ingredients have not yet been isolated and identified, making it difficult to establish the relationship between the two, thus affecting further research in all aspects. So that the current version of the Pharmacopoeia of the People’s Republic of China (2020 edition) has clear quality standards for ST, but it is still not possible to effectively differentiate between forgeries due to its weak specificity and low content limits. There has been a great deal of progress in scientific research on the identification and fingerprinting of indicator components (Liu et al., 2019; Sun et al., 2021). Yet further efforts are needed to incorporate these results into legal quality standards. To establish a more reasonable and effective evaluation system to improve the quality of ST, as well as to find specific ranges of administration to ensure safe and valid clinical medication, thus providing a reference basis for the development and utilization of ST resources.

② Building “brain-heart” disease models are the future development direction. The therapeutic effect of ST on CCVDs fully reflects the TCM principle of “treating the brain and the heart simultaneously”, but it still needs to be verified by constructing a model for both cardio-cerebral vascular diseases, so as to treat both and reduce the pathological damage of cardiac and cerebral tissues at the same time. In addition, the specific potency and strength of ST in compound formulas are unknown, and there are fewer studies on the formulation of effective components. Extensive cross-sectional studies have been conducted on the interventional effects of multiple pathological aspects after cardiovascular and cerebrovascular injury, but there are fewer in-depth longitudinal studies on individual pathological aspects, as well as few studies at the molecular biological level. Therefore, there is a necessity to further explore the biological activity and potential pharmacological mechanism through systems pharmacology approaches, combined with multi-omics tools to screen and predict the targets of active substances and to elucidate their specific pharmacological mechanisms.

③ The homeostatic mechanism of BBB regulation by ST remains to be studied in depth. The special function of the BBB makes the treatment of cerebrovascular diseases difficult because the majority of drugs are difficult to pass through the BBB, making it impossible for drugs to be delivered to the CNS and failing to accumulate in the brain to reach a sufficient dose to affect the efficacy. In recent years, how to promote the efficient opening of the BBB to improve the curative effect in CNS diseases has gradually become a hot research topic. On the one hand, ST can perform neuroprotective effects, and on the other hand, it can open the BBB so that the drug itself and the accompanying drugs can enter the brain parenchyma and directly perform neuroprotective effects. However, it is worrying whether any harmful matter may enter the brain parenchyma along with the drug and aggravate the neurological damage while the BBB is opened by ST. Further investigations into the mechanism and time window for BBB opening are awaited.

④The pharmacokinetic processes of the pharmacodynamic substances in ST need to be explored. So far, there have been relatively few reports on the pharmacokinetics of ST. This sparse report still used HPLC to establish a pharmacokinetic study using cinnamic acid as an indicator for determination. Owing to its multi-component, volatile nature, however, the determination of pharmacokinetic parameters of a single component may not be representative of the actual processes of other components *in vivo*. There are very few toxicological studies on ST. According to the literature (Zhang et al., 2020), ST inhibits human cytochrome P450 enzymes (CYPS), which is a double-edged sword. On the one hand, the combination of ST with some CYP-based drugs with a narrow therapeutic window (e.g. warfarin, digoxin, etc.) may trigger herb-drug interactions, which may inhibit drug metabolism and induce clinical adverse effects or complications. On the other hand, ST may prolong the duration of CYP-based drugs by blocking their metabolic clearance, which is beneficial for CYP-based drugs with relatively rapid clearance rates and thus improves therapeutic efficacy. The active ingredients for these effects are the pentacyclic triterpenes. In summary, the *in vivo* processes of ST need to be systematically understood and studied in depth. Studies on the blood and metabolic components of ST itself and in combination with other drugs will allow for better regulation of the indications and contraindications for clinical use.

In view of the above, the therapeutic effect of ST on CCVDs adequately reflects the TCM treatment principle of “treating both heart and brain”, which is in line with the treatment strategy of holistic medicine and has great potential for development and research value. In the future, further in-depth examinations should be planned on the basis of existed studies to avoid low-level repetition in order to obtain new advancements and breakthroughs.
